# Fall-Related Emergency Department Admission: Fall Environment and Settings and Related Injury Patterns in 6357 Patients with Special Emphasis on the Elderly

**DOI:** 10.1155/2014/256519

**Published:** 2014-03-02

**Authors:** Carmen A. Pfortmueller, Mirco Kunz, Gregor Lindner, Athanasios Zisakis, Stefan Puig, Aristomenis K. Exadaktylos

**Affiliations:** ^1^Department of General Internal Medicine, University Hospital and University of Bern, 3010 Bern, Switzerland; ^2^Department of Emergency Medicine, University Hospital and University of Bern, 3010 Bern, Switzerland; ^3^Division of Anesthesiology, Cantonal Hospital of Winterthur, 8400 Winterthur, Switzerland; ^4^University Institute of Diagnostic, Interventional and Pediatric Radiology, University Hospital and University of Bern, 3010 Bern, Switzerland

## Abstract

*Principals*. Throughout the world, falls are a major public health problem and a socioeconomic burden. Nevertheless there is little knowledge about how the injury types may be related to the aetiology and setting of the fall, especially in the elderly. We have therefore analysed all patients presenting with a fall to our Emergency Department (ED) over the past five years. *Methods*. Our retrospective data analysis comprised adult patients admitted to our Emergency Department between January 1, 2006, and December 31, 2010, in relation to a fall. *Results*. Of a total of 6357 patients 78% (*n* = 4957) patients were younger than 75 years. The main setting for falls was patients home (*n* = 2239, 35.3%). In contrast to the younger patients, the older population was predominantly female (56.3% versus 38.6%; *P* < 0.0001). Older patients were more likely to fall at home and suffer from medical conditions (all *P* < 0.0001). Injuries to the head (*P* < 0.0001) and to the lower extremity (*P* < 0.019) occurred predominantly in the older population. Age was the sole predictor for recurrent falls (OR 1.2, *P* < 0.0001). *Conclusion*. Falls at home are the main class of falls for all age groups, particularly in the elderly. Fall prevention strategies must therefore target activities of daily living. Even though falls related to sports mostly take place in the younger cohort, a significant percentage of elderly patients present with falls related to sporting activity. Falls due to medical conditions were most likely to result in mild traumatic brain injury.

## 1. Introduction

Throughout the world, falls are a major public health problem and a socioeconomic burden [1–6]. Approximately one-third of the population over the age of 65 falls each year, rising to over 50% by the age of 80 [5, 7–10]. Falls and fall-related injuries are a major cause of disability and personal and professional impairment [3, 6–8]. According to the leading Swiss Accident Insurance provider (SUVA), the prevalence of falls in Switzerland is approximately 295,000 cases per year: 55,000 at work, 140,000 during leisure time, and 100,000 at home [11]. These figures have increased in recent years, in particular in the leisure sector [11]. Particularly in the elderly population, falls are associated with increasing costs, due to the greater morbidity in this population [1, 9]. As a consequence, the initiation of fall prevention programs is of high priority [1, 8].

Even though much has been written about fall prevention, less is known about fall environment and settings and the injuries related to them. Emergency department (ED) data can provide valuable information on fall environment and setting, as both inpatient and outpatient data are included. We have therefore assessed all patients presenting with a fall to our Emergency Department in a five-year period. The primary aim of this study is to assess fall environments and settings and related injuries in geriatric patients in comparison to the younger cohort.

## 2. Material and Methods

### 2.1. Data Collection and Retrospective Survey

Our retrospective data analysis comprised adult (≥16 years) patients admitted to our Emergency Department between 1 January 2006 and 31 December 2010 in relation to a fall. A fall was defined as “unintentional coming to rest at ground level or at another lower level” [10]. All patients presenting to the ED with a fall during the study period were initially eligible for study inclusion. They were identified using the appropriate search string in the diagnosis or medical history field of our computerized patient database (Qualicare Office, Medical Database Software, Qualidoc AG, Bern, Switzerland). The following clinical data was extracted from medical records: admission date, aetiology of the fall, localisation of injury, type of injury, anticoagulant medication, hospitalisation, duration of hospitalisation, in-hospital mortality, and numbers of falls during the study period (termed “recurrent falls”). Demographic data, such as gender, age, nationality, and profession, were also assessed. Geriatric patients were defined as being equal to or more than 75 years of age. All medical records were reviewed by an internal specialist, a surgical specialist and a specialist in emergency medicine. Fall environment and related injuries were extracted according to diagnosis and history; no ICD 10 coding was used. The fall environment or setting was categorized into seven classes (home, public space, sport, work, intoxication, medical condition, and other). Each patient was categorized into only one group. All specialists had to agree independently on the classification. Patients with medical fall setting compromised patients presenting with a medical condition (e.g., syncope, seizure, and stroke) and a fall. Patients with falls related to public space fell while ambulating as a pedestrian (including traffic accidents) or in public buildings, such as a railway station. Falls in at home were defined as falls in the house itself or its close surroundings (e.g., in the garden). Patients with the aetiology “others” included all patients involved in some sort of violence (e.g., being pushed or hit). All intoxicated patients were categorized as such. If a patient was intoxicated and fell at home he was classified as intoxication. The same classification pattern was applied to patients suffering from a fall due to a medical condition or others. The location of the injury and the injury pattern were extracted from the diagnosis and radiological imaging. Categorization was performed according to the most serious injury. Multiple injury was defined according to Zelle et al: “≥ 2 severe injuries, with at least one injury or the sum of all injuries being life threatening” [12]. These injuries were categorized as “multiple including the head” or as “multiple without including the head.” Mild traumatic brain injury was defined as GCS >13 with a loss of consciousness for less than 5 minutes, nausea and emesis, or amnesia for the fall. Severe traumatic brain injury was defined as GCS <12. Injuries to the pelvis were categorized as “lower extremity” injuries; injuries to the shoulder girdle (including the clavicle) were classified as “upper extremity” injuries. “Severe injury” was defined as either severe traumatic brain injury or multiple injury. Patients with duplicated records (*n* = 233), a fall in their medical history unrelated to the presentation (*n* = 457) and incomplete records (*n* = 1561) (aetiology of fall not extractable from the medical history), were excluded from the analysis.

### 2.2. Statistical Analysis

All statistical analyses were performed with the SPSS 20.0 Statistical Analysis program (SPSS Inc., Chicago, IL). Descriptive statistics (i.e., numerical, percentage data, means, and standard deviations) were used to analyse most of the data. The significance of the data was analysed with the *χ* ^2^ test (Pearson, Fisher exact test) for qualitative data and the unpaired *t*-test and unidirectional ANOVA (Tamhane-T2) for quantitative data. Correlation was assessed by Spearman's analysis and multivariate linear regression. All *P* values were two tailed and at a level of significance of 0.05.

### 2.3. Ethical Considerations

The study was approved by the Ethics Committee of the Canton of Bern, Switzerland.

## 3. Results

Out of 320,000 ED visits over a five-year study period, a total of 6357 patients were eligible for the study.  Table 1 lists the patient characteristics. 78% (*n* = 4957) patients were younger than 75 years. 509 patients (9.6%) presented with recurrent falls.

**Figure 1:**
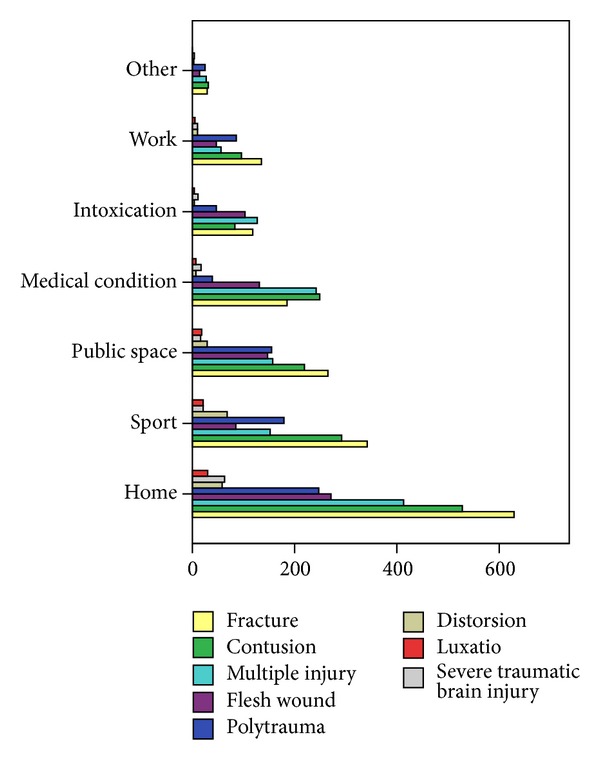
Distribution of injury type.

The main environment or setting for falls was at home (*n* = 2239, 35.3%), followed by sports (*n* = 1160, 18.2%), public space (*n* = 1006, 15.8%), medical conditions (*n* = 877, 13.8%), intoxication (*n* = 497, 7.8%), work (*n* = 445, 7.0%), and others (*n* = 133, 2.1%). For an overview of injury types and related environments and settings see Figure 1. Patients suffering a fall in public space (OR 1.2, *P* < 0.015), a fall at work (OR 1.6, *P* < 0.0001), or an injury during sport (OR 1.2, *P* < 0.004) were at increased risk of severe injury. A fall due to a medical condition is negatively associated with severe injury (OR 0.4, *P* < 0.0001). Multiple injuries were most often caused by falls at home (*n* = 247, 31.7%), followed by falls during sport (*n* = 179, 23.0%) and falls at work (*n* = 155, 19.9%) (see Table 2).

Information on hospitalisation was available for 6030 patients. 57.4% (*n* = 3650) of patients were outpatients and 37.4% (*n* = 2380) inpatients. The mean time in hospital was 6 days (1–132). 4.2% (*n* = 262) of our patients died.

Table 1 summarises the characteristics of the geriatric and younger patients. The median age of geriatric patients was 82.5 (range 75–100). In contrast to the younger patients, the older population was predominantly female (56.3% versus 38.6%; *P* < 0.0001). Older patients were more likely to fall in the house, suffer from medical conditions that resulted in a fall, and to fall due to other conditions (all *P* < 0.0001). In contrast to the geriatric patients, younger patients were predisposed to fall due to sport, in public space, intoxication, or at work (all *P* < 0.0001). Fractures were more common in the elderly (*P* < 0.0001) and were associated with female gender (*P* < 0.0001). Injuries to the extremities in the elderly were associated with increased risk of a fracture (lower extremity: 2.29, CI 1.92–2.52, *P* < 0.0001; upper extremity 2.2, CI 2.18–2.92), but not contusion (OR 0.92, CI 0.69–1.34, *P* < 0.91). Elderly patients with falls due to medical conditions (OR 0.68, CI 0.55–0.85, *P* < 0.0001) were at decreased risk of fractures. For all other fall classes, no significant association with fracture was found. Mild traumatic brain injuries occurred more often in the female elderly (*P* < 0.0001), as did falls due to medical conditions (OR 1.47, CI 1.17–1.74, *P* < 0.001). Multiple injuries in the elderly were most likely to occur due to falls in public space (OR 1.95, CI 1.12–3.43, *P* < 0.027), followed by sports (OR 1.85, CI 1.19–2.78, *P* < 0.011).

Older patients took anticoagulant medication significantly more often (35.3% versus 11.8%; *P* < 0.0001). 58.8% (*n* = 823) of our older patients were hospitalized, compared to 31.4% of the younger patients (*n* = 1557, *P* < 0.0001). Elderly patients were hospitalised for longer (10 versus 8 days, *P* < 0.0001) and were at increased risk of dying after a fall (OR 3.82, 3.02–4.83, *P* < 0.001) (see Table 2 for a risk analysis).

Age was the sole predictor for recurrent falls (OR 1.2, *P* < 0.0001).

## 4. Discussion

We aimed to characterise fall environments and settings and the injury patterns related to them in geriatric patients in comparison to younger patients in an academic teaching hospital ED over a 5-year period. Our results show that geriatric patients compromise 22% of all patients admitted with a fall to our Emergency Department, with more than half experiencing a fall in their home.

Our study shows that falls at home affect all age groups, but are most common in the elderly [3, 6, 13, 14]. Sartini et al. [3] reported slightly lower figures than ours, with 46.3% of all falls occurring at home. These authors also examined activities undertaken at the time of the falls [3]. It showed that individuals aged 65+ years were resting, sleeping, eating, or engaging in other vital activities, whereas younger patients were predominantly involved in leisure activities at the time of fall [6]. This may explain why in our study older patients predominantly fell in the house whereas the younger cohort suffered from fall-related injuries attributed to sports. The increased fall prevalence at home in the elderly may also be related to the fact that elderly people tend to spend more time in the home environment than younger people [15]. Chronic medical conditions such as impaired sight and muscular weakness may deter the elderly from going outside [9, 15–19]. In conclusion, elderly people most often fall in relation to activities of daily living.

Even though falls related to sports are predominant in the younger cohort, our study shows that falls related to sports are the third most common cause of falls in the elderly, a finding that is quite remarkable. In the USA, sport injuries in the elderly (older than 65 years) make up 8% of all sport injuries, with an upward trend [20]. In our study, 9.9% of all patients older than 75 suffered from a sport-related fall. This number is distinctly higher than the findings from the USA. Generally, the increased rates of falls related to sports may be explained with the increasing tendency for people in their 70s or 80s to have a more active lifestyle [20]. Moreover, according to the US National Electronic Injury Surveillance System, one reason that falls in the elderly have increased is that older people now participate more often in more active sports, such as skiing and in-line skating [20]. A number of studies have shown that these activities make high demands on coordination, reaction time, and balance [10, 21, 22], abilities that decrease with age. Nevertheless sport in the elderly also has positive aspects. Studies have shown that moderate exercise programs in the elderly improve muscle strength and balance and may therefore reduce the risk of falling [20, 23].

In our study, mild traumatic brain injury was the second most common type of injury in the elderly and the third most common in the younger cohort. In the elderly, these injuries occurred predominantly at home and in relation to medical conditions. The high occurrence rate of mild traumatic brain injuries in the elderly is confirmed by Watson and Mitchell [2]. The high prevalence of mild traumatic brain injuries in relation to falls related to medical conditions may be caused by the loss of consciousness accompanied by, for example, syncope. Patients therefore might not use their hands to protect their head and in consequence their fall may not be decelerated by the hands as it normally would. Additionally, falls in the elderly may more often result in mild traumatic brain injuries than in the younger population, due to inefficient reflexes and reduced ability to break their falls [2].

In contrast to other studies, elderly patients in our study were less likely to be injured more severely than were younger patients [6, 14, 24]. This may be explained by the fact that we are the only level I trauma centre in a large alpine region. Surprisingly, falls at home, the leading environment of falls in this study, was not correlated with severe injury. Only speculation is possible here. One possible reason may be that falls at home often occur on one single level. Furthermore, falls at home usually do not involve further hazards that may cause additional injury, such as high speed or a collision. The same phenomenon may also explain why falls from medical conditions even correlate negatively with injury severity.

### 4.1. Limitations

Our findings have to be considered with some caution. Information was incomplete or unclear in 1561 patients and these patients had to be excluded. In consequence, underreporting of falls, their environments/settings, and the related injuries cannot be excluded. Additionally our single centre study may not represent the population of all of Switzerland and our results may therefore not be generalizable. The division in to settings and environments may lead to further bias as, for example, some of the patients suffering from intoxication and a fall may have been in the house at the time of the fall. The same applies to the patients with falls due to medical conditions and others. Additionally, it is possible that some of our patients suffered from recurrent falls during the study period that did not require ED treatment or where these patients were treated elsewhere (general practitioner, different ED); it is therefore likely that our figures concerning recurrent falls are underestimated. Furthermore our study was limited to adults (>16 years of age), as children are treated at a separate emergency department in the same hospital. Therefore no information about the epidemiology of falls in children can be drawn from this study. This would require additional studies.

## 5. Conclusion

Falls in the elderly are very common. Falls at home are the main class of falls for all age groups, particularly in the elderly. Fall prevention strategies must therefore target activities of daily living. Elderly patients presenting to the ED with a fall at home should be screened specifically and if needed appropriate action should be taken.

Even though falls related to sports mostly take place in the younger cohort, a significant percentage of elderly patients present with falls related to sporting activity. As ageing is accompanied by a natural loss of musculoskeletal and neurological abilities, it is particularly important to establish appropriate screening and exercise programs. Falls due to medical conditions were most likely to result in mild traumatic brain injury; therefore, these patients should be carefully evaluated.

Overall falls in the elderly are a huge problem; fall aetiologies and settings should always be assessed in the ED and appropriate actions should be taken. Further studies should be performed on how to specifically screen elderly fall patients in the ED.

## Figures and Tables

**Table 1:** Patients characteristics.

	*N* (%)	*N* (%)	*N* (%)	*P* value
	Total	<75	>75	
Demography				
Number	6357 (100)	1400 (22)	4957 (78)	
Mean age (range)	50.9 (16–100)	43.6 (16–74)	82.5 (75–100)	
Men/women	3654/2702 (57.5/42.5)	3042 (61.4)/1914 (38.6)	612 (43.7)/788 (56.3)	0.0001
Fall Environment/setting				
Home	2239 (35.2)	1523 (30.7)	716 (51.1)	0.0001
Public space	1006 (15.8)	925 (18.8)	81 (5.8)	0.0001
Sport	1160 (18.2)	1021 (20.6)	139 (9.9)	0.0001
Work	445 (7.0)	397 (8.0)	48 (3.4)	0.0001
Intoxication	497 (7.8)	460 (9.2)	37 (2.6)	0.0001
Medical condition	877 (13.8)	534 (10.8)	343 (24.5)	0.0001
Others	133 (2.1)	97 (2.0)	36 (2.5)	0.0001
Type of Injury				
Contusion	1498 (26.9)	1235 (24.9)	263 (18.8)	0.0001
Flesh wound	798 (14.2)	615 (12.4)	183 (13.1)	0.52
Distorsion	179 (3.2)	174 (3.5)	5 (0.4)	0.006
Luxation	85 (1.5)	69 (1.4)	16 (1.1)	0.59
Fracture	1703 (30.5)	1275 (25.7)	428 (30.6)	0.0001
Mild traumatic brain injury	1174 (21.0)	843 (17.0)	331 (23.6)	0.0001
Severe traumatic brain injury	142 (2.5)	93 (1.9)	49 (3.5)	0.001
Multiple injury	780 (12.3)	653 (13.2)	127 (8.9)	0.0001
Severe Injury	922 (14.5)	746 (15.0)	176 (12.4)	0.014
Anticoagulation	1083 (17.0)	589 (11.8)	494 (35.3)	0.0001
Hospitalisation				
Hospitalisation rate	3650 (57.4)	1557 (31.4)	823 (58.8)	0.0001
Mean hospitalisation duration (days)	6 (1–132)	8 (1–132)	10 (1–115)	0.001
In-hospital mortality	262 (4.1)	126 (2.5)	136 (9.7)	0.0001
Recurrent fall	509 (9.6)	409 (8.3)	200 (14.3)	0.0001

**Table 2:** Risk analysis.

	OR	95% CI	*P* value
Risk of severe injury			
Age < 75	1.04	1.01–1.08	0.01
Age > 75	0.84	0.73–0.97	0.01
House	0.9	0.8–1.1	0.31
Public space	1.2	1.1–1.4	0.015
Sport	1.2	1.1–1.4	0.004
Work	1.6	1.3–2.0	0.0001
Intoxication	0.7	0.6–1.1	0.07
Others	0.4	0.3–0.5	0.0001
Medical condition	0.4	0.3–0.5	0.0001
Hospitalisation rate			
Age < 75	0.6	0.56–0.64	0.0001
Age > 75	1.67	1.58–1.76	0.0001
Risk of in-hospital mortality			
Age < 75	0.92	0.91–0.94	0.0001
Age > 75	3.82	3.02–4.83	0.0001
Risk of recurrent fall			
Age < 75	0.64	0.6–0.7	0.0001
Age > 75	1.2	1.1–1.3	0.0001
Gender	1	0.9–1.3	0.39
